# Reorganized brain functional network topology in stable and progressive mild cognitive impairment

**DOI:** 10.3389/fnagi.2024.1467054

**Published:** 2024-11-18

**Authors:** Chen Xue, Darui Zheng, Yiming Ruan, Xuan Cao, Xulian Zhang, Wenzhang Qi, Qianqian Yuan, Xuhong Liang, Qingling Huang

**Affiliations:** ^1^Department of Radiology, The Affiliated Brain Hospital of Nanjing Medical University, Nanjing, Jiangsu, China; ^2^Division of Statistics and Data Science, Department of Mathematical Sciences, University of Cincinnati, Cincinnati, United States

**Keywords:** progressive mild cognitive impairment, stable mild cognitive impairment, rich club, module, graph theory

## Abstract

**AIM:**

Mild cognitive impairment (MCI) includes two distinct subtypes, namely progressive MCI (pMCI) and stable MCI (sMCI). The objective of this study was to identify the topological reorganization of brain functional networks in patients with pMCI and sMCI.

**Methods:**

Resting-state functional magnetic resonance imaging (rs-fMRI) was applied to patients with pMCI, sMCI and healthy controls. Graph theory was applied to study the topological characteristics of the brain’s functional networks, examining global and nodal metrics, modularity, and rich-club organization. Analysis of covariance and two sample t-tests were applied to assess differences in topological attributes between patient groups, alongside correlation analysis, which examined the value of changing topological attributes in predicting various clinical outcomes.

**Results:**

Significant differences between each group with regard to network metrics were observed. These included clustering coefficients and small-worldness. At the nodal level, several nodes with an abnormal degree centrality and nodal efficiency were detected. In rich club, pMCI and sMCI patients showed declined connectivity compared with HC. Significant differences were observed in the intra- and inter-module connections among the three groups. Particularly noteworthy was the irreplaceable role of the cerebellar module in network interactions.

**Conclusion:**

Our study revealed significant differences in network topological properties among sMCI, pMCI and HC patients, which were significantly correlated with cognitive function. Most notably, the cerebellar module played a crucial role in the overall network interactions. In conclusion, these findings could aid in the development of imaging markers used to expedite diagnosis and intervention prior to Alzheimer’s disease onset.

## Introduction

Alzheimer’s disease (AD) is a neurodegenerative disorder characterized by progressive cognitive decline and memory impairment (Silva et al., 2019). Amnestic mild cognitive impairment (aMCI) is a largely asymptomatic antecedent of AD. However, given the progressive nature of this cognitive impairment, intervening at this pre-clinical stage is critical in preventing eventual AD onset. Patients with aMCI fall into two categories. Progressive MCI (pMCI) is defined as aMCI, which progressively develops into AD. Meanwhile, stable MCI (sMCI) is defined as aMCI, which either remains stable or improves over time (Chen et al., 2023; Qian et al., 2018). Currently, clinical biomarkers of AD primarily include changes in levels of tau proteins (total tau and phosphorylated tau) and β-amyloid 1–42 peptide in cerebrospinal fluid (Botello-Marabotto et al., 2023). However, due to their invasiveness, obtaining these biomarkers can be burdensome to elderly patients who are already affected by the disease.

In recent years, Resting-state functional magnetic resonance imaging (rs-fMRI) has developed rapidly, offering high temporal and spatial resolution that allows for the detection of spontaneous neuronal activity in brain networks during the resting state (Sun et al., 2023). This technology has become increasingly important in studying brain function, contributing to a more comprehensive understanding of network changes in the brain during the progression of AD (Jiang et al., 2018). At the same time, graph theory has emerged as a popular method for describing the characteristics of brain networks. In this approach, the human brain is considered as a highly intricate network that can be modelled using a collection of nodes representing distinct brain regions and a series of edges representing the connections between these regions (Bertolero et al., 2018). rs-fMRI brain network methods have been widely used to identify important topological features in human brain functional networks through graph theory analysis (Wang et al., 2013; Zdanovskis et al., 2021). In light of the growing evidence that suggested that network connectivity may help predict future Alzheimer’s disease (AD) diagnosis, analyzing differences in brain network connectivity might provide insights into distinguishing between patients with sMCI and pMCI (Delorme and Makeig, 2004; Stevens and Kircher, 1998). Several studies of brain graph theory have confirmed that brain connections are not uniformly distributed. Instead, significantly higher numbers of connections appear in certain nodes of the brain, a phenomenon referred to as “rich club” (van den Heuvel and Sporns, 2011; Yan et al., 2018; Zamora-Lopez et al., 2010). These nodes exert a significant influence on the brain’s network topology and are closely associated with global information integration (Xu et al., 2010; Yan et al., 2018). In addition, a wealth of evidence has shown the brain to be organized into distinct, specialized communities, a phenomenon known as “modularity”. Neurons within each of these communities or “modules” have stronger intra-module connectivity compared to inter-module connectivity, promoting efficient information processing (Bertolero et al., 2015; Bertolero et al., 2017; Meunier et al., 2010). Modularity and rich club, as well as global and nodal connectivity indicators, such as the small-world characteristic and nodal effectivity, play different roles in network communication. Studying these indicators can help reveal distinct and unique patterns in the connectome of the brain.

Rich club nodes have been suggested to have higher metabolic demands compared to other nodes, potentially making them more susceptible to the impact of degenerative diseases (Bullmore and Sporns, 2012). The existing body of research suggests that the global and local network organization of the whole brain is altered in AD and antecedent cognitive decline (Li et al., 2018; Shu et al., 2012). Patients with each stage of AD (from preclinical to late-stage dementia) have distinctive distributions of centrally concentrated lesions (Buckner et al., 2009; Crossley et al., 2014). Recently, researchers have investigated the predictive efficacy of various measures of modularity in assessing MCI and AD progression stage (de Haan et al., 2012). Previous studies using multiparametric graph theoretical analysis revealed altered functional and structural network topology in AD and these different connectivity metrics indicated additional or complementary information of brain networks regarding the topological changes in MCI or AD (Berlot et al., 2016; Jiang et al., 2018). Compared with other methods focusing on functional network connectivity and brain network properties in patients with cognitive impairment, whether differences in multiparametric network may be predictive of a patient having either pMCI or sMCI is unknown. Studying the alterations in brain network properties associated with pMCI and sMCI is crucial for understanding the patterns of changes in their brain networks and elucidating the pathological mechanisms underlying both conditions.

Therefore, in this study, we used rs-fMRI to construct functional networks for sMCI, pMCI, and healthy control (HC) patients. Subsequently, we used graph theory analysis to compare the diversity of the topological properties of the whole-brain functional networks across three groups. Our main goal was to explore the differences in the network topological properties between pMCI and sMCI. Overall, we hypothesized: 1) pMCI and sMCI network topology attributes to be different; 2) differences to potentially be significantly associated with cognitive function; 3) during the progression of AD, alterations in specific brain regions may occur, which might contribute to the diagnosis and prediction of the disease.

## Methods

### Participants

The applied research data for our study were acquired from the Alzheimer’s disease Neuroimaging Initiative (ADNI) database.^1^ The details regarding the diagnostic criteria used to categorize patients into either pMCI, sMCI or HC groups are provided in Supplementary Table 1.

### MRI data acquisition

We attained all MRI scans on a 3.0T scanner, unifying scanning protocols obtained from various manufacturers, including Philips (Best in the Netherlands), General Electric (Cleveland, OH, USA) and Siemens (Munich, Germany). Detailed information can be obtained from the MRI Training Manual FINAL.pdf^2^ and the http://adni.loni.usc.edu/wp-content/uploads/2017/07/ADNI3-MRI-protocols.pdf.

### Neuropsychological assessment

Participants’ general cognitive abilities were evaluated by the Montreal Cognitive Assessment (MOCA). Meanwhile, episodic memory (EM) was assessed using the composite score derived from the Rey Auditory Verbal Learning Test, the Alzheimer Disease Assessment Scale-Cognitive, Logical Memory and the mini mental state exam (MMSE) and executive function (EF) was assessed using the composite score derived from Category Fluency, WAIS-R Digit Symbol, Trails A & B, DigitSpan Backwards, and clock drawing tests. All neurocognitive assessments are available on the ADNI website.^3^ The methods for measuring EM and EF are described in the Supplementary Table 1.

### Data pre-processing

The preprocessing was conducted in MATLAB (2015b) and Data Processing and Analysis for Brain Imaging (DPABI), which was based on the Statistical Parametric Mapping software package (SPM12). The details regarding image pre-processing are provided in the Supplementary Table 1.

### Network construction

Functional connectivity networks were analysed using the Graph Theoretical Network Analysis (GRETNA) toolbox (Wang et al., 2015). The details regarding network construction are provided in the Supplementary Table 1.

### Network properties

Recent researches have suggested that small-world topology exists in functional brain networks (Xue et al., 2020). To research the topological attributes of each network, the study assessed the following graph metrics (see Supplementary Table 1): characteristic path length (Lp), normalized characteristic path length (λ), clustering coefficient (Cp), normalized clustering coefficient (γ), small-world parameters (σ), global efficiency (Eg), local efficiency (Eloc), betweenness centrality (BC), degree centrality (DC), nodal efficiency (NE), and nodal local efficiency (NLE). Additionally, for each property, we calculated the area under the curve, providing a scalar, which was independent of threshold selection. This allowed better characterization of the topological characteristics of the brain network.

### Modular organization

Based on previous research (Suo et al., 2022), the AAL116 template divided the 116 regions of interest (ROIs) into six modules, namely the sensorimotor network (SMN), default mode network (DMN), frontoparietal network (FPN), visual network (VN), subcortical network (SN), and cerebral network (CN). A modularity metric, Q, was calculated to assess the degree of subdivision within each network into specific modules, which were defined by having more intra-modular connections than inter-modular connections (Newman, 2006). The GRETNA software utilized a modified greedy optimization algorithm to identify the optimal modular architecture by averaging the functional networks of all participants. For each subject, the mean intra-modular strength was defined as the average number of connections to other regions of the selected module, whilst the mean inter-modular strength was defined as the average number of connections between the selected module and other modules.

### Rich-club organization

Rich-club regions were defined as the top 13 regions with the highest average nodal degree of all regions in HC patients, accounting for 12% of the total number of regions (Daianu et al., 2015; Yan et al., 2018). Regions other than the rich-club regions were classified as peripheral nodes. The edges in the network were categorized into three types of connections: rich-club connections, which linked two rich-club nodes; feeder connections, which connected one rich node and one peripheral node; and local connections, which connected two peripheral nodes (van den Heuvel et al., 2013). The connectivity strength was a summary measure of connectivity, which was calculated using the sum of the edge weights for each connection type (Yan et al., 2018).

### Statistical analysis

Analysis of covariance (ANCOVA) and chi-squared tests were used to compare the demographic and neurocognitive data across the three groups, containing pMCI, sMCI and HC patients. Bonferroni’s correction with a *p* < 0.05 was used for post hoc analysis when test parameters were met.

Furthermore, two-sample *t*-tests (age- and sex-corrected) were performed to compare the AUC values of network metrics, including between the three groups (*p* < 0.05, FDR-corrected).

Lastly, correlation analysis was conducted to explore the relationship between altered network metrics and various types of cognitive function, including EM and EF. The correlation between network metrics and age, gender, years of education and volume of grey matter was also assessed (Bonferroni-corrected, *p* < 0.05).

All statistical tests and comparisons were conducted using the Statistical Package for the Social Sciences (SPSS; version 22.0; IBM, Armonk, NY, USA).

## Results

### Demographic and clinical variables

Table 1 presents the demographic and neurocognitive characteristics of all participants, including 31 pMCI, 41 sMCI, and 82 HC participants. SPSS revealed that the HC group exhibited a significant difference in years of education compared to the pMCI and sMCI groups and significant differences in cognitive performance were noted between all groups. SPSS revealed that the pMCI and sMCI groups exhibited significantly lower EM, EF, and MOCA test scores compared to the HC group (Bonferroni’s post hoc correction, *p* < 0.05). Detailed neuropsychological test results are provided in Supplementary Table 1.

**TABLE 1:** Demographics and clinical measures of three groups, including pMCI, sMCI, and HC.

	pMCI (31)	sMCI (40)	HC (80)	*F*-values (χ ^2^)	*P*-values
Age (years)	72.99 (7.06)	71.46 (7.68)	72.68 (6.02)	0.593	0.554
Gender (F/M)	15/16	19/21	46/34	0.863	0.650
PTEDUCAT	15.60*	15.71*	17.04	5.594	0.005^ac^
MMSE	26.93 (1.78)***/*	27.90 (1.54)***	29.06 (1.39)	23.874	<0.001^abc^
MOCA	21.50 (3.79)***/*	23.76 (3.30)***	26.23 (2.72)	27.347	<0.001^abc^
EM	−0.11***/***	0.43***	1.07	54.720	<0.001^abc^
EF	−0.08***/**	0.57**	1.16	27.259	<0.001^abc^

Numbers are given as means (standard deviation, SD) unless stated otherwise. Scores reflect the number of correct items unless stated otherwise. Values for age derived from ANOVA; gender from chi-square test; all clinical measures from ANOVA with age and gender as covariates. MMSE, Mini-Mental State Examination; MOCA, Montreal Cognitive Assessment; EM, episodic memory; EF, executive function; a, *post-hoc* analyses showed a significantly group difference between pMCI and HC; b, *post-hoc* analyses showed a significantly group difference between pMCI and sMCI; c, *post-hoc* analyses showed a significantly group difference between sMCI and HC;

*p < 0.05;

**p < 0.01;

***p < 0.001; pMCI, progressive mild cognitive impairment; sMCI, stable mild cognitive impairment; HC, healthy controls; Detailed neuropsychological test results are provided in SI results.

### Group differences in global network organization

**FIGURE 1:**
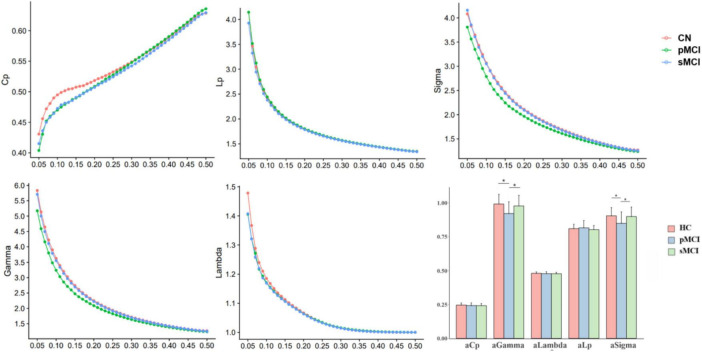
The differences in global metrics of the brain functional networks among progressive mild cognitive impairment, stable mild cognitive impairment, and healthy controls. pMCI, progressive mild cognitive impairment; sMCI, stable mild cognitive impairment; HC, healthy controls; Cp, clustering coefficient; Lp, shortest path length; sigma (δ), small-world characteristic; gamma (γ), normalized clustering coefficient; lambda (λ), normalized characteristic path length; aCp, AUC in Cp; aGambda, AUC in Gambda; aLambda, AUC in Lambda; aLp, AUC in Lp; aSigma, AUC in Sigma. *p < 0.05.

In this experiment, HC, pMCI and sMCI patients all showed small-worldness (γ > 1, λ ≈ 1, σ > 1.1). Cp Eloc and Eglob for all three groups increased with higher thresholds, while the values of Lp, γ, λ, and σ decreased (Figure 1). Interestingly, as shown in Figure 1, compared with pMCI patients, those with sMCI or who were HCs had significantly lower values of γ and σ (FDR < 0.05).

### Group differences in nodal network metrics

**FIGURE 2:**
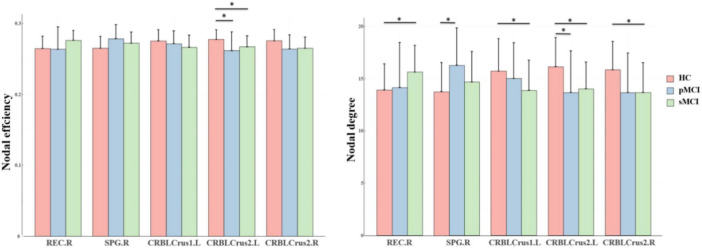
The bar graphs show the post hoc pairwise comparisons with significant differences in nodal efficiency and nodal degree. The y-axes are the area under the curve of the two network parameters and the three groups are color coded as in the key. The x-axis shows the brain regions. pMCI, progressive mild cognitive impairment; sMCI, stable mild cognitive impairment; HC, healthy controls; REC.R, right gyrus rectus; SPG.R, right superior parietal gyrus; CRBLCrus1.L, left superior cerebellum; CRBLCrus2.L, left inferior cerebellum; CRBLCrus2.R, right inferior cerebellum; *p < 0.05.

As shown in Figure 2, for nodal metrics, compared with HC patients, pMCI showed significantly increased Dc in the right superior parietal gyrus (SPG.R) and decreased Dc and Ne in the left inferior cerebellum (CRBLCrus2.L). Meanwhile, sMCI patients showed significantly increased Dc in the right gyrus rectus (REC.R) and decreased Dc in right (CRBLCrus2.R) and the left inferior cerebellum, and left superior cerebellum (CRBLCrus1.L) compared with HC patients. No significant differences in these metrics were observed between patients in the pMCI and sMCI groups.

### Group differences in intra-and inter-modular connections

**FIGURE 3:**
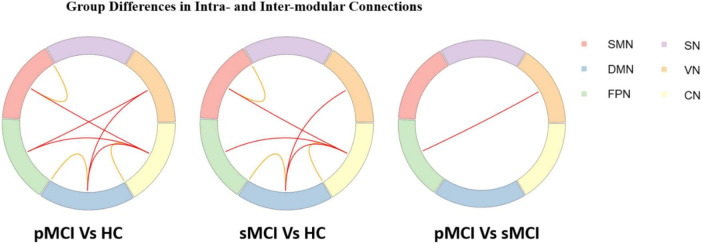
Progressive mild cognitive impairment, stable mild cognitive impairment, and healthy controls have significant differences in intra- and inter-modular connections. pMCI, progressive mild cognitive impairment; sMCI, stable mild cognitive impairment; HC, healthy controls; SMN, sensorimotor network; DMN, default mode network; FPN, frontoparietal network, VN, visual network; SN, subcortical network; CN, cerebral network.

As shown in Figure 3, for inter-modular connections, pMCI patients showed increased connectivity between the SN and CN, the DMN and VN, the FPN and VN, and the FPN and CN when compared with HC patients. Meanwhile, sMCI patients showed increased connectivity between the CN and SMN, the FPN and DMN and the SMN and DMN when compared with HC patients. Importantly, compared with pMCI patients, sMCI showed decreased connectivity between the FPN and VN. For intra-modular connectivity, compared with HC patients, both sMCI and pMCI patients showed decreased connectivity within the SMN, DMN and CN. Although there was no significance between pMCI and sMCI patients with regard to intra-modular connectivity, pMCI patients did display a non-significant downward trend.

### Group differences in rich-club organization

**FIGURE 4:**
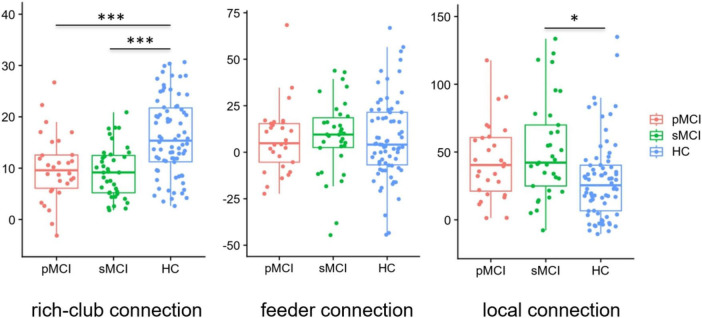
Boxplots exhibit connectivity strengths for rich club, feeder and local. pMCI, progressive mild cognitive impairment; sMCI, stable mild cognitive impairment; HC, healthy controls; *p < 0.05, ***p < 0.001.

As shown in Figure 4, based on the group-averaged functional network, the rich-club nodes were defined as the top 13 (12%) brain regions with the highest average nodal degree of all regions in HC patients. For rich-club connection, there was a significant difference between HC patients and both pMCI and sMCI patients (*p* < 0.05). For local connection, there are significant differences between sMCI and HC. However, there was no significant difference for feeder connection.

### Correlation analysis

**FIGURE 5:**
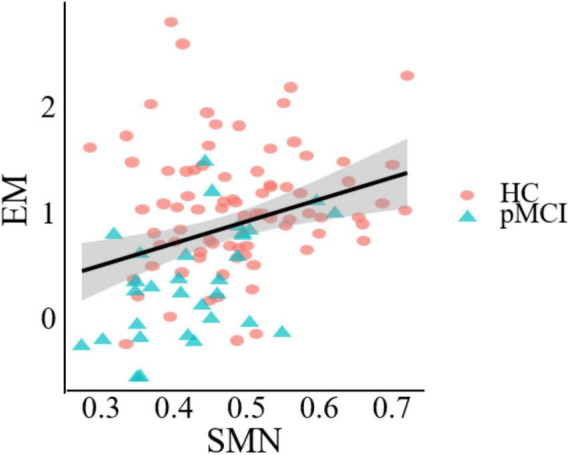
Significant associations between altered network metric and cognitive function in groups between pMCI and HC. Age, the volume of gray matter and sex were used as covariates of results (Bonferroni-corrected, *p* < 0.05). EM, episodic memory; SMN, sensorimotor network.

Figure 5 demonstrates a significant correlation between network topology attributes and neurocognitive test scores for patients with pMCI and HC. The intramodular connectivity in SMN was positively correlated with EM (*p* = 0.0152, *r* = 0.4622).

## Discussion

We employed graph theory analysis in the study with rs-fMRI data to explore if they exist in differences of the network topology across pMCI, sMCI, and cognitive health. Meanwhile, whether the whole-brain functional network could serve as predictors of the three patient groups. Consistent with our hypotheses, our research found in the following: first, there were significant differences among the pMCI, sMCI, and HC patient groups in terms of global properties, local properties, rich club organization, and modularity. Moreover, these differences were significantly associated with cognitive function. Lastly, our findings indicated that these network topological properties were most strongly associated with the CN. Understanding the value of the CN in AD severity may provide valuable information for analyzing pathology mechanisms and predicting the prognosis of MCI patients.

Global and local properties are related to the small-world property, which is measured by the degree to which networks exhibit high clustering coefficients and short path lengths (Sun et al., 2014). Networks with such properties were thought to possess higher network and local efficiency, resulting in faster information transmission (van den Heuvel et al., 2008). The previous studies revealed the pMCI and sMCI patient groups exhibited some special small-world topology (Sun et al., 2018). In our study, compared with both HC and sMCI patients, pMCI patients showed significantly lower small-world parameters (σ), and normalized clustering coefficient (γ). γ was defined as the probability of connections between adjacent regions, and its reduction in pMCI patients may suggest disruptions in brain networks, leading to restricted information flow (Pereira et al., 2016). Based on a previous study, this limitation may impact memory, cognition and other cognitive functions that may be linked to the progression to dementia in AD (Li et al., 2012). At the same time, networks with a high small-world parameter (σ) have most nodes tightly connected, while maintaining a short average path length between any two nodes, enabling efficient local information processing and rapid global communication (Guo et al., 2022). A decrease in σ may indicate that the integration and allocation of the pMCI networks have been affected. These reduced global network attributes indicated the functional network of pMCI patients to be abnormal with reduced efficiency of specialized and integrated processing (van den Heuvel et al., 2008; Wang et al., 2013). Moreover, in comparison to pMCI patients, our findings indicated sMCI patients to not exhibit a significant decline in global properties. This could be attributed to the actions of compensatory mechanisms in these individuals, which might effectively offset impaired brain function. Ultimately, similar to previous studies, our results showed that various small-world topological properties change with the progression of AD, and the differing degrees of change between pMCI and sMCI may help distinguish between them (Sanz-Arigita et al., 2010; Xue et al., 2020).

Nodal network metrics may contribute to distinguish from the three groups in this study, and we found that compared with HC patients, pMCI and sMCI patients exhibited significantly lower degree centrality (Dc) and nodal efficiency (Ne) at CRBLCrus1.L, CRBLCrus2.L, CRBLCrus2.R, while the opposite was at SPG.R, REC.R. Degree centrality is an important attribute measuring the number of connections a node has with other nodes its own network, while nodal efficiency is an index to evaluate the efficiency of information transmission (Itahashi et al., 2014; Liao et al., 2017). Our results implied higher hub connectivity to be associated with increased vulnerability to pathology, likely because such connectivity would have been more energy intensive to maintain (Griffa and Van den Heuvel, 2018). Alteration in the network properties of these nodes may affect the connectivity and efficiency of communication with other areas more. Therefore, early prevention of the decline of these hub nodes connections may delay the course of AD.

The rich club refers to a trend in brain networks where high-degree nodes are more tightly connected than low-degree nodes, with these hub nodes (rich club nodes) connections playing a crucial role in global information transmission (Albert et al., 2000; Xue et al., 2019). In our study, compared with HC patients, there were significantly decreased rich-club connections in both pMCI and sMCI patients. Additionally, as mentioned above, we found that reduced node attributes in the disease group were mainly within the rich club nodes, while increased attributes were primarily located outside these nodes. In a previous study, it had been demonstrated that AD spectrum rich-club connection is preferentially attacked (Shu et al., 2018), which was consilient with our findings. Besides, although there was no significant difference between sMCI and pMCI patients with regard to the number of feeder and local connections, we found that connections tended to be higher in pMCI than in sMCI patients. This elevation of non-richclub connections may compensate for some of the impaired brain function caused by reduced rich-club connections. However, the rich-club connections were a leading factor in facilitating the comprehensive integration of neural information across diverse brain regions, explaining why global information processing function in pMCI and sMCI patients was still reduced.

In contrast to the high-degree nodes of the rich club, modularity refers to groups of nodes within a network that are densely connected internally but sparsely connected externally (Liang et al., 2015). These modular groups achieve a complex balance between energy costs and communication efficiency, and changes in their structure can impact system efficiency and performance (Andric and Hasson, 2015; Sporns and Betzel, 2016). In our study, both pMCI and sMCI patients had widespread alterations compared with HC patients at the modular level. They showed decreased intra-modular connectivity within the DMN, SMN and CN. The DMN is considered to be a key network in several neurodegenerative diseases, including AD (Buckner et al., 2008; Turner and Spreng, 2012). A decline in intra-modular connectivity within the DMN in disease groups may lead to reduced efficiency in information transmission within the network. Moreover, recent studies have also shown certain regions of the SMN to play an important role in the regulation of memory (Liang et al., 2013). Consistent with this, the results of this study indicated a significant correlation between intra-module connectivity of the SMN and EM test scores, revealing the important role of this network in perceptual cognition, motor learning, and other aspects of MCI disease progression (Figure 5). Interestingly, both pMCI and sMCI patients showed an upward trend in inter-modular connectivity compared to HC patients, possibly to counter the cognitive decline caused by the decline in intra-modular connectivity. At the same time, we found pMCI patients to have a significantly higher number of connections between the FPN and VN compared to sMCI patients. This is consistent with previous studies showing that cognitive decline in aMCI patients is related to disrupted connectivity between the FPN and VN and that this disruption accelerates progression to AD (Chen et al., 2022). Patients with pMCI may compensate for their declining cognitive function by increasing connectivity.

Most importantly, our experiment revealed that changes in both intra-modular and inter-modular connectivity were predominantly associated with CN, suggesting that CN may play an indispensable role in the progression of AD. In the past, most researches on AD focuses on the interaction of cerebral networks, the thought of an effect of cerebellar networks on AD may be a new perspective. Previous studies have shown that cognitive dysfunction after CN injury involves multiple cognitive domains (Yao et al., 2022). Additionally, changes in the modular structure in aMCI were primarily observed in the CN (Zhang et al., 2020). In fact, in our study, we observed that nodes with significantly different attribute values at the nodal level and hub nodes within the rich club predominantly reside within the CN. Additionally, in terms of modularity, the CN exhibited the highest degree of interaction with other network modules. Damage to cerebellar networks can lead to extensive disconnection of whole brain networks (Wang et al., 2022). Our study has contributed to new evidence indicating abnormalities in CN connectivity among individuals with MCI. Furthermore, our research highlights the importance of understanding the relationship between CN connectivity and MCI, given the potential for this knowledge to potentially help delay the progression of the disease. By gaining a deeper understanding of these connections, we can develop more effective interventions and treatments to improve outcomes for individuals with early-stage cognitive decline.

## Limitations

There were several limitations to the current study. First, the patient sample size was small, perhaps making the results less generalizable. However, to avoid this problem, we applied a non-parametric permutation test to improve the accuracy and will continue to increase our sample size as the ADNI database is updated. Second, the HC showed significant differences according to years of education in the pMCI and sMCI groups. Thus, years of education were treated as a covariable in all the analyses.

## Conclusion

Our study revealed significant differences in network topological properties among the three groups of participants, which were significantly correlated with cognitive function. Furthermore, distinct patterns of connectivity were observed within and between modules. Most notably, the cerebellar module played a crucial role in overall network interactions. This suggested that exploring the progression of Alzheimer’s disease with a focus on cerebellar networks to be a potentially viable strategy. In conclusion, these findings may be used as imaging markers for early diagnosis and intervention of AD.

## Data Availability

The original contributions presented in the study are included in the article/Supplementary material, further inquiries can be directed to the corresponding authors.

## Footnotes

1
www.adni-info.org

2
http://adni.loni.usc.edu/wpcontent/uploads/2010/05/ADNI2

3
https://ida.loni.usc.edu/pages/access/studyData
