# Supplementary material for: Reorganized brain functional network topology in stable and progressive mild cognitive impairment
Source: Front Aging Neurosci. 2024 Nov 18;16:1467054. doi: 10.3389/fnagi.2024.1467054 (PMC11609165; doi:10.3389/fnagi.2024.1467054)
Supplement: Supplementary file 1 [file Table_1.doc]

***SI***

***Methods***

**Participants**

ADNI is a longitudinal multiterm study designed to develop clinical, imaging, genetic, and biochemical biomarkers for the early detection and tracking of AD. In our investigation, patients with MCI evolving into AD within 4 years were defined as progressive mild cognitive impairment (pMCI), while those who remained unchanged or improved were defined as stable mild cognitive impairment (sMCI). We extracted rs-fMRI data from 170 participants, including 96 healthy controls (HC) participants and 42 participants with sMCI and 32 with pMCI. Additionally, 19 individuals were excluded due to excessive head motion, defined as cumulative translation or rotation > 3.0 mm or 3.0◦. Consequently, the final dataset included 151 participants, consisting of 80 CN, 40 sMCI, and 31 pMCI participants.

**Image preprocessing**

All fMRI data were preprocessed by MATLAB 2015b and Data Processing and Analysis for Brain Imaging (DPABI), which is based on Statistical Parametric Mapping (SPM12). Data pre-processing included the following steps: removal of the first 10 volumes to enhance precision of the images, and the rest performed slice-timing correction and head motion correction. The participants with excessive head motion (cumulative translation or rotation of > 3. mm or 3.0) were excluded[1, 2]. Then, spatially normalizing images to the Montreal Neurological Institute (MNI) echo-planar imaging template and resampled to 3 × 3 × 3 mm^3^ voxels; Following this, nuisance variables, such as 24 head motion parameters, global mean signal, white matter signal, and cerebrospinal fluid signal, were severally removed to reduce the effect on the dependent variable[3, 4]. Subsequently, the normalized brain volumes were smoothed by Gaussian kernel of 6 × 6 × 6 full widths at half maximum in order to reduce individual variations[5]. Finally, to control noise interferences such as heartbeat and breathing, we selected a filtering frequency of 0.01–0.08Hz.

For the global network metrics, we quantified the characteristic path length (L_p_), normalized characteristic path length (λ), clustering coefficient (C_p_), normalized clustering coefficient (γ), small-world parameters (σ), global efficiency (E_g_), and local efficiency (E_loc_). For the regional characteristics, we considered the nodal clustering coefficient, nodal shortest path length, and betweenness centrality. All network analyses were performed using the GRETNA software (<http://www.nitrc.org/projects/gretna/>). Detailed definitions of the network metrics and nodal metrics are provided as follows.

**Network Construction**

To define network nodes, the brain was divided into 116 regions of interest (ROIs) using the automated anatomical labeling (AAL) atlas (Tzourio-Mazoyer et al., 2002). Pearson correlation coefficients were calculated between the mean time series of each pair of ROIs, representing the network edges. This resulted in a binary correlation matrix of size 116×116 for each participant. To ensure consistency across groups, a comprehensive range of sparsity values from 0.05 to 0.50 with a step size of 0.01 were applied to the correlation matrices when computing network metric, ensuring that the number of edges was equivalent among the three groups. Furthermore, negative correlations were excluded from all functional brain networks due to their ambiguous interpretation and their negative impact on test-retest reliability.

**Network Properties**

**Network efficiency**. The global efficiency of G measures the global efficiency of the parallel information transfer in the network, which can be computed as:


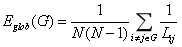


Where L_ij_ is the shortest path length between node i and node j in G.

The local efficiency of G shows how efficient the communication is among the first neighbors of the node i when it is removes and reveals how much the network is fault tolerant [6]. The local efficiency of a graph is defined as:


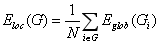


Where G_i_ denotes the subgraph composed of the nearest neighbors of node i.

**Small-world properties**. Small-world network parameters mainly include characteristic path length (L_p_), normalized characteristic path length (λ), clustering coefficient (C_p_), normalized clustering coefficient (γ), and small-world parameters (σ). In the study, we investigated the small-world properties of the binary brain networks.

The path length between node i and node j is defined as the sum of the edge lengths along the path. The shortest characteristic path length plays an important role in the information transmission and communication of network. The shortest path length, L_ij_, is defined as the length of the path for node i and node j with the shortest length. The shortest path length was computed as follows:


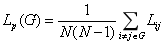


Where N is the number of nodes in the network.

The clustering coefficient of a node i, C(i), which was defined as the possibility whether the neighborhoods were connected with each other or not, was computed as follows:


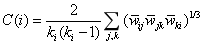


where k_i_ is the degree of node i and
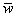
 is the weight of edge, which is scaled by the maximum weight of the network. The clustering coefficient, C_p_, of the network is the average of the clustering coefficient over all nodes and indicates the degree of local interconnection or clustering in the network. If the nodes are isolated or have just one connection, i.e., k_i_ = 0 or k_i_ = 1, the clustering coefficient is zero.

To examine the small-world properties, the characteristic path length (L_p_), and clustering coefficient (C_p_) of the networks were compared with those of random networks. In the present study, we generated 1000 matched random network with the same number of nodes, edges, and degree distribution as the real networks. Furthermore, we computed the normalized characteristic path length,
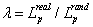
 and the normalized clustering coefficient,
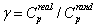
, where
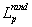
 and
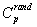
are the averaged L_p_ and the averaged C_p_ of 1000 matched random networks. If the studied network has a high clustering coefficient (
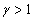
) and short characteristic path length (
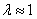
) relative to the random network, then the network has the property of “small world” [7]. Humphries et.al combined these two measurements (γ and λ) into a simple quantitative metric, small-wordness,
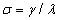
, to measure the “small world” property [8]. When σ >1, the network has the “small world” property.

**Regional nodal characteristics**. To determine the nodal characteristics of the brain networks, we computed the nodal degree, nodal efficiency, nodal clustering coefficient, nodal shortest path length, and betweenness centrality.

The nodal degree, k_nodal_, defined as the number of edges connected to the node, is a fundamental basic network measure to assess the central role of a region in brain networks. A node with a great nodal degree is classified as a hub that is highly connected to other nodes. The nodal degree was computed as follows:


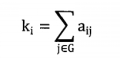


where a_ij_ means there is a directly connection between node i and node j in G.

The nodal efficiency, E_nodal_, is defined as:


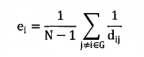


where d_ij_ is the shortest path length between node i and node j in G. Node efficiency reflects the ability of a node to transmit information to other nodes in the network.

The betweenness centrality, B_nodal_, is defined as:


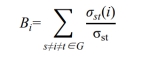


where
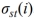
is the number of shortest path length from node s to node t. The betweenness centrality measures the importance of node i in G as a bridge.

**Neuropsychological Assessment**

ADNI-EM: ADNI adopted a single factor model for data analysis, dividing the dataset into two parts: one with annual visits data (baseline, months 12 and 24) and another with additional visits (months 6, 18, and 36). In the first part, Logical Memory and three MMSE items, along with the first RAVLT version, were used as anchors, with variations due to different ADAS-Cog versions. A longitudinal model was applied to the annual visit data, setting the general factor's variance to 1 at baseline and allowing its mean and variance to vary over time. Indicator loadings on the general factor were consistently estimated across time points. This method yielded precise estimates for memory assessment loadings and thresholds. For the second dataset part, the second RAVLT version and other anchors were used to estimate this RAVLT version's parameters, employing a similar modeling strategy to the first part. This ensured the scale remained anchored to the baseline's established metrics, allowing for free estimation of means and variances at each visit. ADNI calculated the ADNI-MEM (memory) factor scores for participants at all study visits up to 36 months. For the 48-month neuropsychic battery, which contained identical memory items as the baseline, baseline parameters were reused to compute 48-month scores, ensuring consistency and utilizing the model's capacity to monitor memory performance longitudinally.

ADNI-EF: ADNI developed the ADNI-EF model using baseline data through an iterative process involving confirmatory factor analysis, small group reviews, and model revisions, utilizing Mplus software with specific settings. The final ADNI-EF model included a range of executive function (EF) indicators such as Category Fluency tests, Trails A and B, Digit Span Backwards, WAIS-R Digit Symbol Substitution, and Clock Drawing tasks. These indicators, featuring diverse response formats, posed challenges for creating summary measures. To address this, ADNI converted raw scores into ordered categorical scales up to 10 levels, based on the empirical distributions, aiming to preserve variability at the distribution tails. This approach facilitated the development of composite scores without assuming specific score distributions.

***Results***

**Table1: Neurocognitive Characteristics**

|  | pMCI | sMCI | CN | Fvaluse((χ 2) | P values |
| --- | --- | --- | --- | --- | --- |
| ADAS11 | 13.06(4.946)***/*** | 8.71(3.321) | 8.20(3.321) | 17.740 | <0.001ab |
| ADAS13 | 20.42(7.200)***/*** | 13.84(6.263) | 11.83(5.310) | 22.552 | <0.001ab |
| ADASQ4 | 6.23(2.501)***/** | 4.51(2.405)*** | 2.70(2.147) | 28.048 | <0.001abc |
| RAVLT_immediate | 29.83(7.625)***/* | 35.90(8.735)*** | 47.64(10.531) | 44.709 | <0.001abc |
| RAVLT_learning | 3.34(2.176)***/* | 4.90(2.683) | 5.95(2.687) | 11.044 | <0.001ab |
| RAVLT_forgetting | 5.55(2.308)*** | 4.79(1.922)** | 3.00(2.716) | 14.550 | <0.001ac |
| RAVLT_perc_forgetting | 75.23(24.963)***/** | 55.18(23.627)*** | 28.67(28.591) | 36.567 | <0.001abc |
| LDELTOTAL | 4.57(3.371)***/** | 7.55(2.806)*** | 14.02(3.873) | 92.899 | <0.001abc |
| TRABSCOR | 142.41(77.925)***/*** | 92.05(36.493)** | 69.83(35.719) | 25.352 | <0.001abc |
| FAQ | 7.07(4.713)***/*** | 1.87(2.745)* | 0.30(1.757) | 61.161 | <0.001abc |
| Aβ | 663.89** | 864.06 |  | 0.779 | <0.001ac |
| p-tau | 33.80* | 25.02 |  | 1.93 | 0.16 |
| t-tau | 339.83 | 267.69 |  | 0.223 |  |

Numbers are given as means (standard deviation, SD). Scores reflect the number of correct items unless stated otherwise. ADAS: Alzheimer Disease Assessment Scale-Cognitive; ADASQ4: ADAS Delayed Word Recall; RAVLT, Rey Auditory Verbal Learning Test; LDELTOTAL, the longitudinal effect of APOE-ɛ4 genotype on the logical memory delayed recall total; TRABSCOR, Trail Making Test Part B Time; FAQ, Functional Activities Questionnaire; Aβ, amyloid‑β protein; t-tau, total tau protein;p-tau, phosphorylated tau; *, p < 0.05; **, p < 0.01;***, p < 0.001; pMCI, progressive mild cognitive impairment; sMCI, stable mild cognitive impairment; CN, cognitively normal;

**References**

1. Lei, B., et al., *Diagnosis of early Alzheimer's disease based on dynamic high order networks.* Brain Imaging Behav, 2021. **15**(1): p. 276-287.

2. Gu, Y., et al., *Abnormal dynamic functional connectivity in Alzheimer's disease.* CNS Neurosci Ther, 2020. **26**(9): p. 962-971.

3. Niu, H., et al., *Abnormal dynamic functional connectivity and brain states in Alzheimer's diseases: functional near-infrared spectroscopy study.* Neurophotonics, 2019. **6**(2): p. 025010.

4. Dong, G., et al., *Dynamic network connectivity predicts subjective cognitive decline: the Sino-Longitudinal Cognitive impairment and dementia study.* Brain Imaging Behav, 2020. **14**(6): p. 2692-2707.

5. Cordova-Palomera, A., et al., *Disrupted global metastability and static and dynamic brain connectivity across individuals in the Alzheimer's disease continuum.* Sci Rep, 2017. **7**: p. 40268.

6. Achard, S. and E. Bullmore, *Efficiency and cost of economical brain functional networks.* PLoS Comput Biol, 2007. **3**(2): p. e17.

7. Watts, D.J. and S.H. Strogatz, *Collective dynamics of 'small-world' networks.* Nature, 1998. **393**(6684): p. 440-2.

8. Humphries, M.D., K. Gurney, and T.J. Prescott, *The brainstem reticular formation is a small-world, not scale-free, network.* Proc Biol Sci, 2006. **273**(1585): p. 503-11.
